# Selective memory and behavioral alterations after ambient ultrafine particulate matter exposure in aged 3xTgAD Alzheimer’s disease mice

**DOI:** 10.1186/s12989-019-0323-3

**Published:** 2019-11-26

**Authors:** Katrina Jew, Denise Herr, Candace Wong, Andrea Kennell, Keith Morris-Schaffer, Günter Oberdörster, M. Kerry O’Banion, Deborah A. Cory-Slechta, Alison Elder

**Affiliations:** 1 Department of Environmental Medicine, University of Rochester School of Medicine & Dentistry, Rochester, NY 14642 USA; 2 Department of Neuroscience and Del Monte Neuroscience Institute, University of Rochester School of Medicine & Dentistry, Rochester, NY 14642 USA; 3 Department of Neurology, University of Rochester School of Medicine & Dentistry, Rochester, NY 14642 USA

**Keywords:** Ultrafine particles, Air pollution, Learning, Memory, Neurodegeneration, Alzheimer’s disease, Inhalation exposure, Behavioral testing

## Abstract

**Background:**

A growing body of epidemiological literature indicates that particulate matter (PM) air pollution exposure is associated with elevated Alzheimer’s disease (AD) risk and may exacerbate AD-related cognitive decline. Of concern is exposure to the ultrafine PM (UFP) fraction (≤100 nm), which deposits efficiently throughout the respiratory tract, has higher rates of translocation to secondary organs, like brain, and may induce inflammatory changes. We, therefore, hypothesize that exposure to UFPs will exacerbate cognitive deficits in a mouse model of AD. The present study assessed alterations in learning and memory behaviors in aged (12.5 months) male 3xTgAD and non-transgenic mice following a 2-week exposure (4-h/day, 4 days/week) to concentrated ambient UFPs using the Harvard ultrafine concentrated ambient particle system (HUCAPS) or filtered air. Beginning one month following exposure, locomotor activity, spatial learning and memory, short-term recognition memory, appetitive motivation, and olfactory discrimination were assessed.

**Results:**

No effects on locomotor activity were found following HUCAPS exposure (number concentration, 1 × 10^4^–4.7 × 10^5^ particles/cm^3^; mass concentration, 29–132 μg/m^3^). HUCAPS-exposed mice, independent of AD background, showed a significantly decreased spatial learning, mediated through reference memory deficits, as well as short-term memory deficits in novel object recognition testing. AD mice displayed diminished spatial working memory, potentially a result of olfactory deficits, and short-term memory. AD background modulated HUCAPS-induced changes on appetitive motivation and olfactory discrimination, specifically enhancing olfactory discrimination in NTg mice. Modeling variation in appetitive motivation as a covariate in spatial learning and memory, however, did not support the conclusion that differences in motivation significantly underlie changes in spatial learning and memory.

**Conclusions:**

A short-term inhalation exposure of aged mice to ambient UFPs at human-relevant concentrations resulted in protracted (testing spanning 1–6.5 months post-exposure) adverse effects on multiple memory domains (reference and short-term memory) independent of AD background. Impairments in learning and memory were present when accounting for potential covariates like motivational changes and locomotor activity. These results highlight the need for further research into the potential mechanisms underlying the cognitive effects of UFP exposure in adulthood.

## Introduction

Alzheimer’s disease (AD) is the most prevalent form of dementia and affects around 1 in 8 individuals over the age of 65 years [1]. AD results in progressive and irreversible neurological alterations which impact cognition, quality of life, and lifespan [2]. Even in mild AD cases, extensive damage has already occurred in the brain at the time of diagnosis, where neuronal death causes changes in overall structure and function, most notably within hippocampus and neocortical areas [1, 3]. The clinical hallmark of AD is worsening of two or more cognitive domains, commonly observed in episodic memory and executive functions [4, 5]. Some of the earliest alterations that may be predictive of conversion of mild cognitive impairment (MCI) to AD are olfactory dysfunction [6] and impairments in working and semantic memory [4], which is comparable to reference memory in rodents [7].

The most common form of AD is sporadic AD, occurring later in life than familial AD. While inherited genes, like the apolipoprotein E ε4 allele, can predispose an individual to developing sporadic AD, genes are not the sole contributors to AD risk [1, 8]. One of the proposed risk factors for sporadic AD is air pollution exposure, which is both ubiquitous and already well established as a risk factor for other detrimental health outcomes, namely elevated cardiovascular and pulmonary disease morbidity and mortality [9, 10]. A growing body of epidemiological literature suggests that exposure to elevated concentrations of air pollution is also associated with adverse central nervous system (CNS) outcomes [11–13]. Studies that examine sources of pollution and its constituents have found that elevated exposure to particulate matter (PM) is associated with increased hospitalizations for AD and dementia [11] and with diminished cognitive function [14] and accelerated cognitive decline in the elderly [15]. These findings implicate a role for PM exposure in the progression of AD-related pathology and associated cognitive decline.

Air pollution is a heterogeneous mixture of gaseous and particulate components with high temporal and spatial variability. This makes the identification of causal neurotoxic constituents very difficult [16]. Despite this uncertainty, the ultrafine particulate matter (UFP) fraction of air pollution, consisting of particles ≤100 nm in aerodynamic diameter, is thought to be of particular concern. UFPs are more numerous in the ambient air than larger-sized particles (comprising ~ 80% of all PM by number) [17, 18], have a higher surface area to mass ratio (providing a greater interface for reactivity) [19], and deposit efficiently within all regions of the respiratory tract [20]. It has been observed with laboratory-generated nanoparticles (< 100 nm) that smaller sized particles have prolonged retention in the lung [21] and have a higher potential for translocation across the lung-blood barrier as compared to larger particles [22, 23]. Potential routes of UFP translocation into the CNS include direct retrograde translocation from the olfactory mucosa along olfactory neurons [24–26] or via the trigeminus [27, 28], which extends sensory neurons throughout the nasal mucosa. UFP induced inflammation, systemically and/or within the brain, has been shown to promote AD-related cognitive decline [29, 30] and pathology [31–33]. This makes understanding the possible effects of UFP exposure of growing importance, especially as it has the potential to exacerbate the growing burden of AD upon society [34].

We hypothesized that exposure to concentrated ambient UFP air pollution can exacerbate AD-related cognitive and memory phenotypes. To test this hypothesis, we exposed male 3xTgAD and non-transgenic (NTg) mice to UFPs using the Harvard ultrafine concentrated ambient particle system (HUCAPS) or filtered air (FA) beginning at 12.5 months of age. This is an age during which amyloid-β (Aβ) plaques, hyperphosphorylated-tau tangle pathology, and cognitive alterations are present in male 3xTgAD mice [35, 36]. The 3xTgAD mice are a useful model because they display both the pathological hallmarks and neuroanatomical progression of early human AD stages. Additionally, the male mice of the strain have a well characterized behavioral progression in which spatial learning deficits are reported to initiate at ~ 6 months of age as measured by Morris water maze testing [37]. We performed a battery of behavioral assessments over the course of the ensuing 6.5 months to assess the effects of exposure on locomotor activity, spatial learning and memory, short-term memory, food motivation (progressive ratio schedule), and olfactory discrimination (Table 1).

**Table 1:** Order of behavioral testing

Psychological domain	Behavioral test	Average testing age^a^
Locomotor activity	Spontaneous locomotor activity (SLA)	14 months
Spatial memory	Radial arm maze (RAM)	15.5 months
Short-term object recognition memory	Novel object recognition (NOR)	17 months
Motivation	Progressive ratio schedule (PR)	18 months
Olfaction	Olfactory discrimination	19 months

^a^Average age of the mice during the testing duration

## Results

### Exposure characterization

**Fig. 1:**
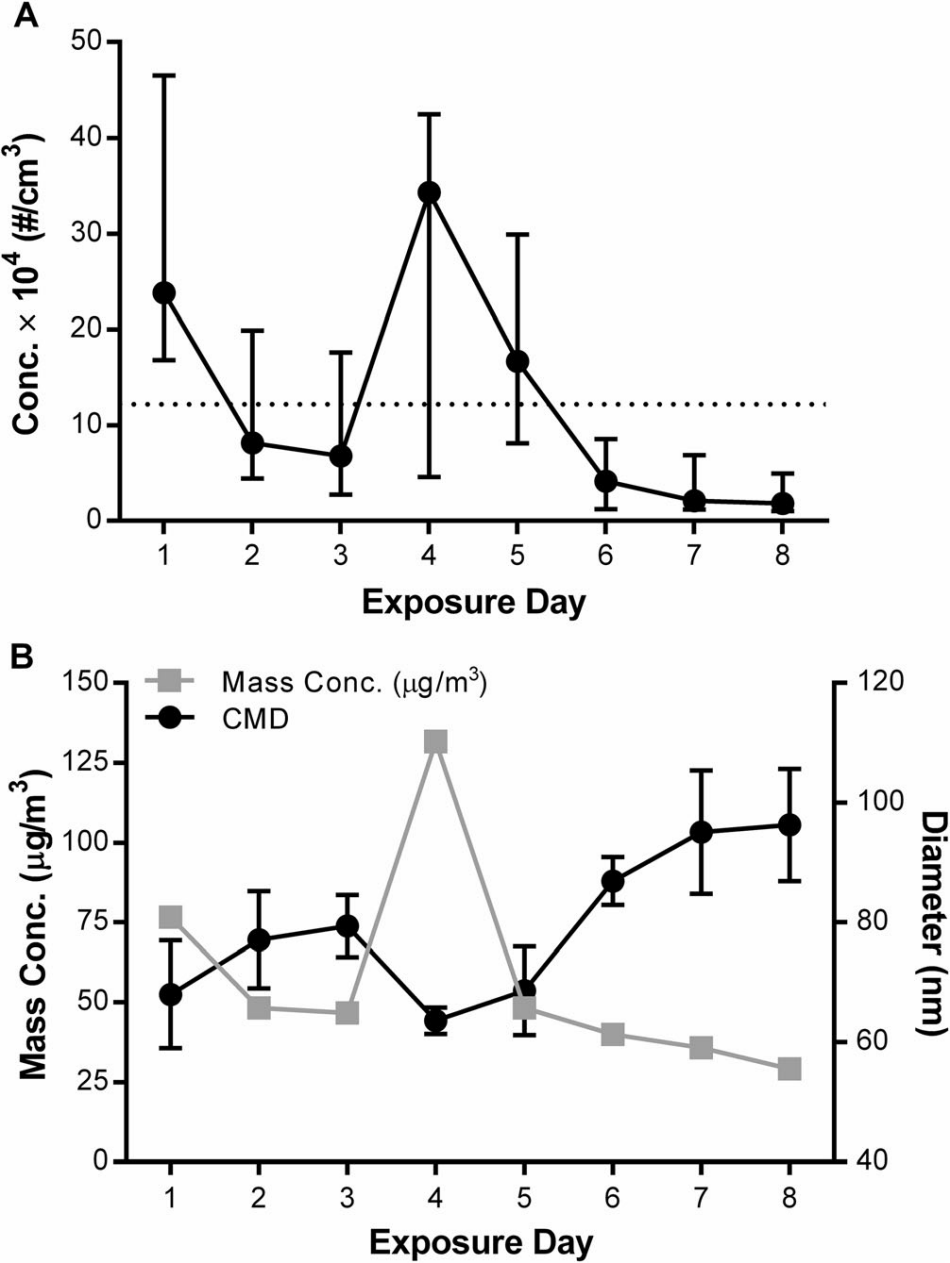
Exposure Characteristics: **a** The average and minimum and maximum particle number concentration (×10^4^ particles/cm^3^) of HUCAPS aerosol per day of exposure. The dotted line denotes the mean number concentration of all exposure days. **b** Gravimetrically determined mass concentration and count median diameter (CMD) (nm) ± standard deviation (SD) per day of exposure

The mean particle number concentration of the HUCAPS aerosol across all exposure days was 122,000 particles/cm^3^ (range, 10,100–465,000 particles/cm^3^) with a mean mass concentration of 57 μg/m^3^ (range, 29–132 μg/m^3^) (Fig. 1). The mean count median diameter (CMD) of the HUCAPS aerosol across all exposure days was 79 nm (range, 64–96 nm) with a geometric standard deviation (GSD) of 1.5, confirming that the aerosol size was narrowly distributed and within the UFP range. Using Multiple-Path Particle Dosimetry (MPPD) modeling [38], we estimated the fractional deposition for the head (15%), tracheobronchial (5%), and pulmonary (20%) regions of the respiratory tract for the aerosol. Using these deposition fractions, we estimated the total daily deposited dose to be 267 ng, with 99 ng deposited on the nasal epithelium, 36 ng within the tracheobronchial region, and 132 ng within the pulmonary region.

### Spontaneous locomotor activity (SLA)

**Fig. 2:**
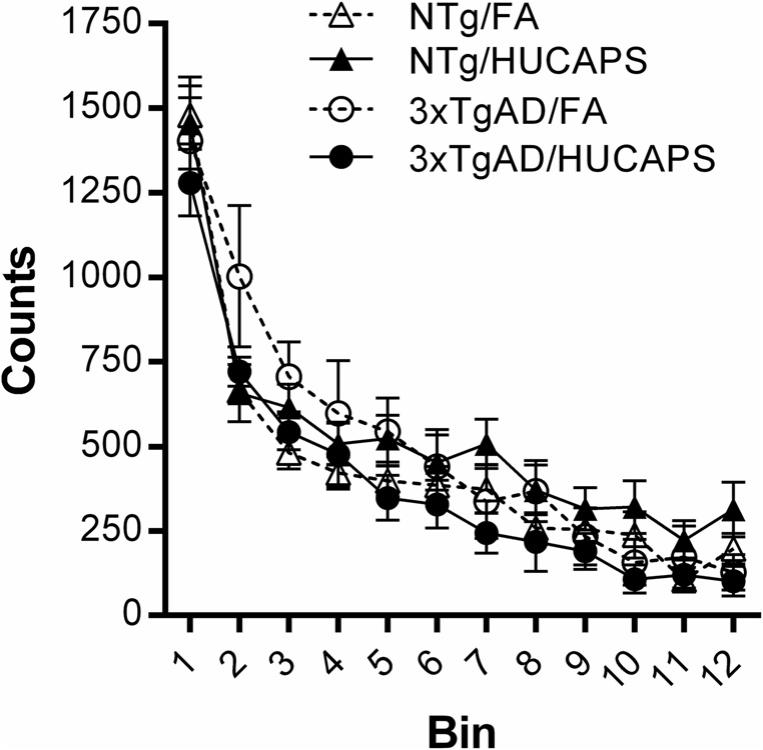
Spontaneous Locomotor Activity: Beam break counts for ambulatory activity as mean ± standard error (SE) aggregated into 5 min epochs (bins) for a total trial time of 60 min. One NTg FA mouse was identified as an outlier by Grubbs’ test and visual inspection and excluded from analysis. *n* = 10–11 per group

There were no statistically significant main effects of genotype or HUCAPS exposure or interactions on ambulatory counts upon SLA testing (Fig. 2).

### Radial arm maze (RAM): spatial learning

**Fig. 3:**
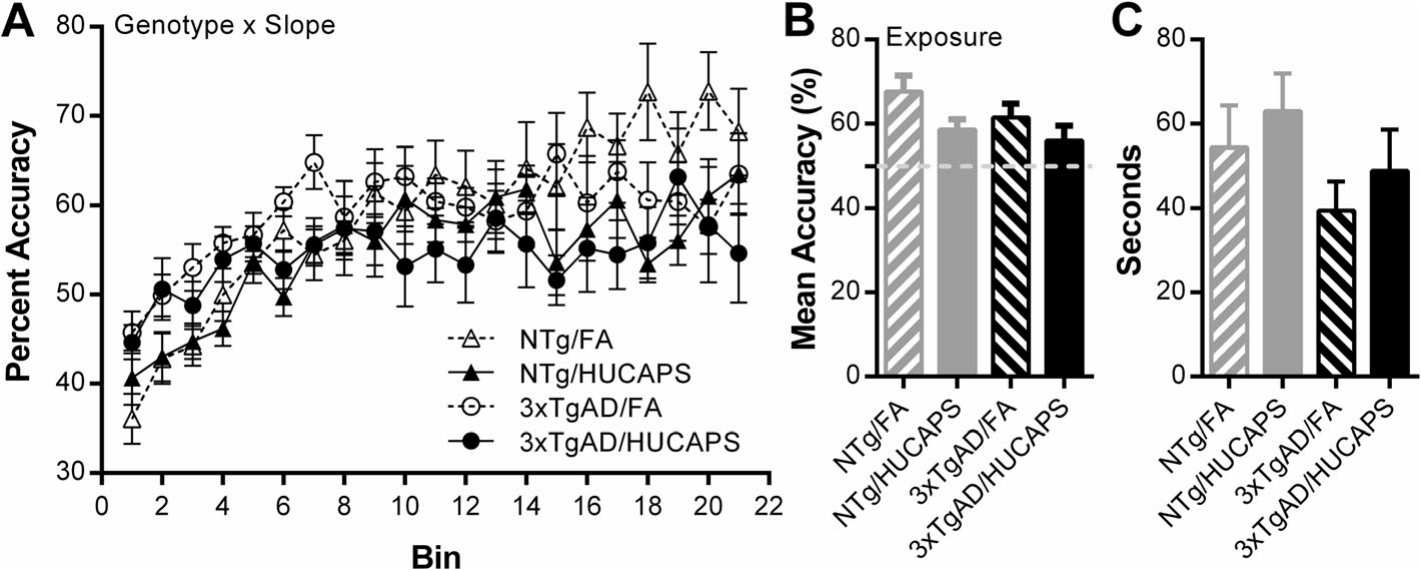
Overall RAM Spatial Learning: **a** Mean percent accuracy across bins of 3 consecutive sessions with the exception of bin 21, which is the average of 4, for a total of 64 sessions. **b** Mean percent accuracy from last 25 tested sessions, session 40 (bin 14) to end of testing. Dashed line shows 50% accuracy. **c** Mean time per arm visit was determined from all tested sessions. Means ± SE. Only significant results are reported at top of the figure. *n* = 10–11 per group

3xTgAD mice had significantly diminished overall rates of spatial learning (flatter slope) compared to NTg mice (β = − 0.234, SE = 0.071, *p* = 0.002) (Fig. 3a; Additional file 1: Supplemental 1). No effect of HUCAPS exposure was found on the overall rates of spatial learning as determined by percent accuracy analysis. There was no significant interaction between genotype and HUCAPS exposure on rate of spatial learning.

No significant main effect of genotype or interactions between genotype and HUCAPS exposure were found on the mean percent accuracy across the final 25 (Fig. 3b). HUCAPS-exposed mice showed a reduced final overall accuracy level as compared to FA-exposed mice (β = − 3.63, SE = 1.65, *p* = 0.034) as assessed by the mean percent accuracy of the final 25 sessions. The final 25 sessions were chosen for analysis as bin 14 is approximately the point in which a divergence of the NTg FA group can be observed and the 3xTgAD HUCAPS mice appear to plateau.

One-sample t-tests were performed on the mean accuracy from the last 25 sessions of each group to determine whether each group performed significantly above chance (50%) as we suspect that a floor affect may be hindering the ability to detect further worsening of spatial learning in the HUCAPS exposed 3xTgAD mice. The NTg FA (*p* = 0.001), NTg HUCAPS (*p* = 0.003), and 3xTgAD FA (*p* = 0.003) groups performed significantly above chance levels of accuracy, whereas the 3xTgAD HUCAPS group did not reach significance.

There were no significant effects of genotype or exposure (or interaction) on the amount of time the mice spent per arm, consistent with the absence of locomotor differences between groups (Fig. 3c).

### Radial arm maze: reference memory

**Fig. 4:**
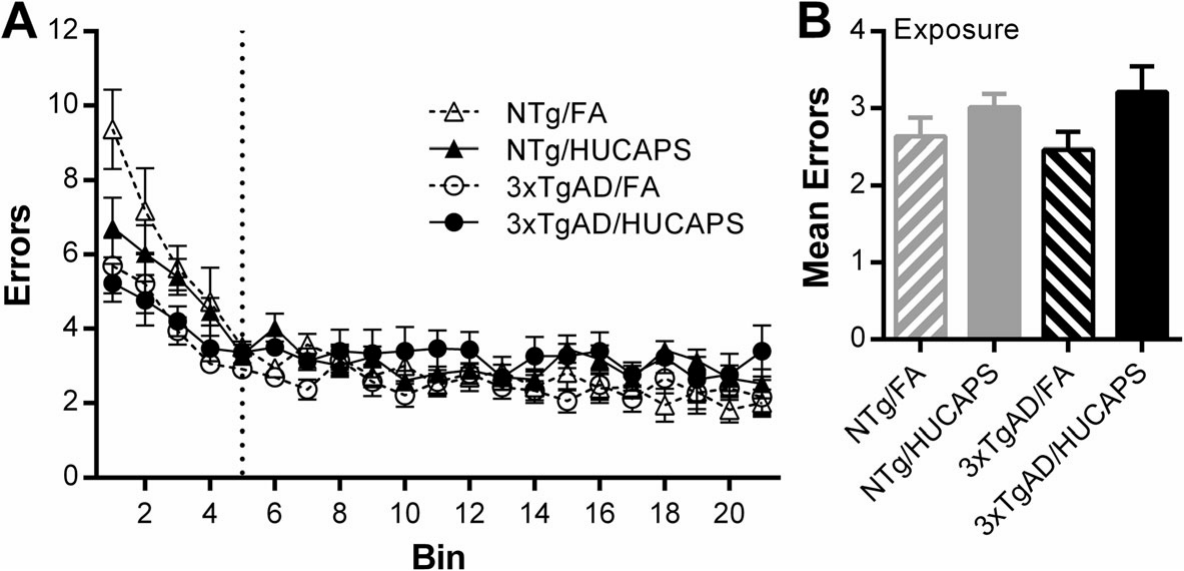
RAM Reference Memory Errors: **a** Reference memory error analysis was split into acquisition phase (AP) and performance phase (PP) with approximate session division shown by dotted vertical line. Graph shows bins representing the mean errors from 3 consecutive sessions with the exception of bin 21, which is the average of 4, for a total of 64 sessions. Analysis of raw session data explored the slope component of each phase. **b** Mean reference memory errors of all performance phase sessions. Means ± SE. Only significant results are reported at top of the figure. *n* = 10–11 per group

Reference memory errors were analyzed separately during an acquisition phase (sessions 1–13) and performance phase (sessions 14–64) of testing (Additional file 1: Supplemental 2). No significant effects of genotype, HUCAPS exposure, or interaction between the two were found on the slope of either the acquisition phase or the performance phase of reference memory errors (Fig. 4a)

No significant effect of genotype or interaction between genotype and exposure was found on mean number of errors across the performance phase sessions (Fig. 4b) (Additional file 1: Supplemental 3). However, a significant effect of exposure was found on the mean number of performance phase errors, where HUCAPS-exposed mice had more errors compared to FA mice (β = 0.282, SE = 0.123, *p* = 0.028). Together this indicates that while HUCAPS did not significantly affect the rate of learning, it did diminish overall reference memory in the performance phase.

### Radial arm maze: working memory

**Fig. 5:**
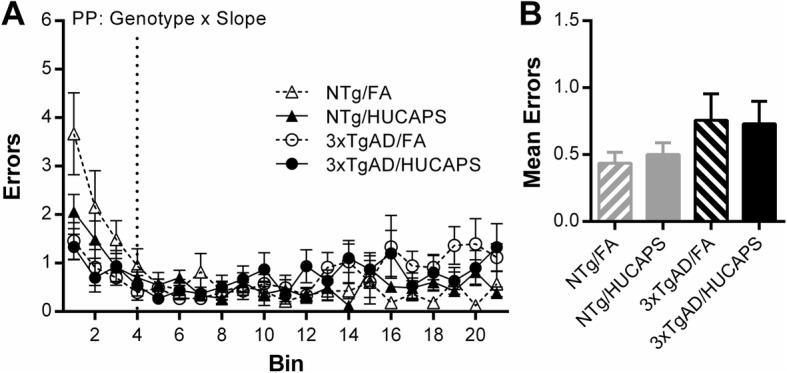
RAM Working Memory Errors: **a** Working memory error analysis was split into acquisition phase (AP) and performance phase (PP) with approximate session division shown by dotted vertical line. Graph shows bins representing the mean errors from 3 consecutive sessions with the exception of bin 21, which is the average of 4, for a total of 64 sessions. Analysis of raw session data specifically explored the slope component of each phase. **b** Mean working memory errors of all performance phase sessions. Means ± SE. Only significant results are reported at top of the figure. *n* = 10–11 per group

Working memory errors were analyzed separately during the acquisition phase (sessions 1–11) and performance phase (sessions 12–64) (Additional file 1: Supplemental 4). In the acquisition phase of working memory, no significant effects of genotype, exposure, or interactions between the two were found on the slope component (error rate) (Fig. 5a). In the performance phase, a significant effect of genotype was found on slope, with 3xTgAD mice exhibiting a diminished rate of memory performance (increasing slope/errors) compared to NTg mice (β = 0.020, SE = 0.006, *p* = 0.002). There were no significant effects of HUCAPS exposure or interaction between genotype and exposure on the slope in the performance phase.

A genotype effect, comparing 3xTgAD and NTg mice mean performance phase errors, approached significance (β = 0.138, SE = 0.071, *p* = 0.060) (Fig. 5b). No exposure related effect or interaction between genotype and exposure was found. Together these points indicate that 3xTgAD spatial learning rate deficits were driven through working memory dysfunction that was detectable within the performance phase.

### Novel object recognition (NOR) testing

**Fig. 6:**
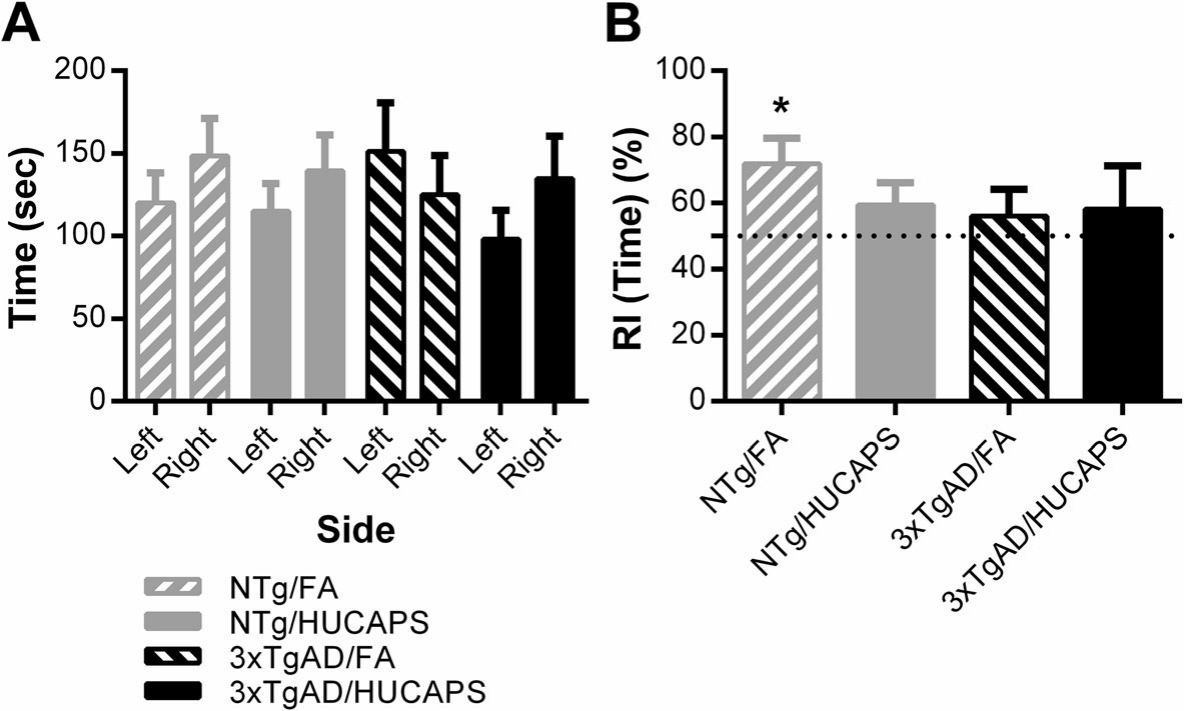
NOR Performance: **a** Total time spent with identical left or right object in session 1. **b** Calculated recognition indices (RI) were derived from time measures in session 2. The dotted line represents 50% RI or recognition due to chance, above which indicates preference for the novel object. Bars depict group mean ± SE values. * indicates significant difference from chance preference in one sided t-test to all fictive groups. *n* = 10–11 per group

In session 1, in which identical stimulus objects are presented, the total time spent with either the right or left object did not significantly differ in relation to genotype, exposure, or chamber side, nor were any interactions detected, suggesting that there was no spatial- or activity-level bias that might confound session 2 interpretation and that the groups spent a similar total amount of time exploring the objects (Fig. 6a).

The recognition index calculated from time measures, RI (Time), found that only the NTg FA group (*p* = 0.027) demonstrated a significant preference for the novel object in session 2 upon comparison to 6 fictive datasets generated from a hypothetical population centered around μ = 50% to test novel preference (see Discussion and Methods for more information) (Fig. 6b) (Additional file 1: Supplemental 5). There were no significant effects (or interaction) in RI (Time) by 2-factor ANOVA**.**

### Progressive ratio (PR) schedule

**Fig. 7:**
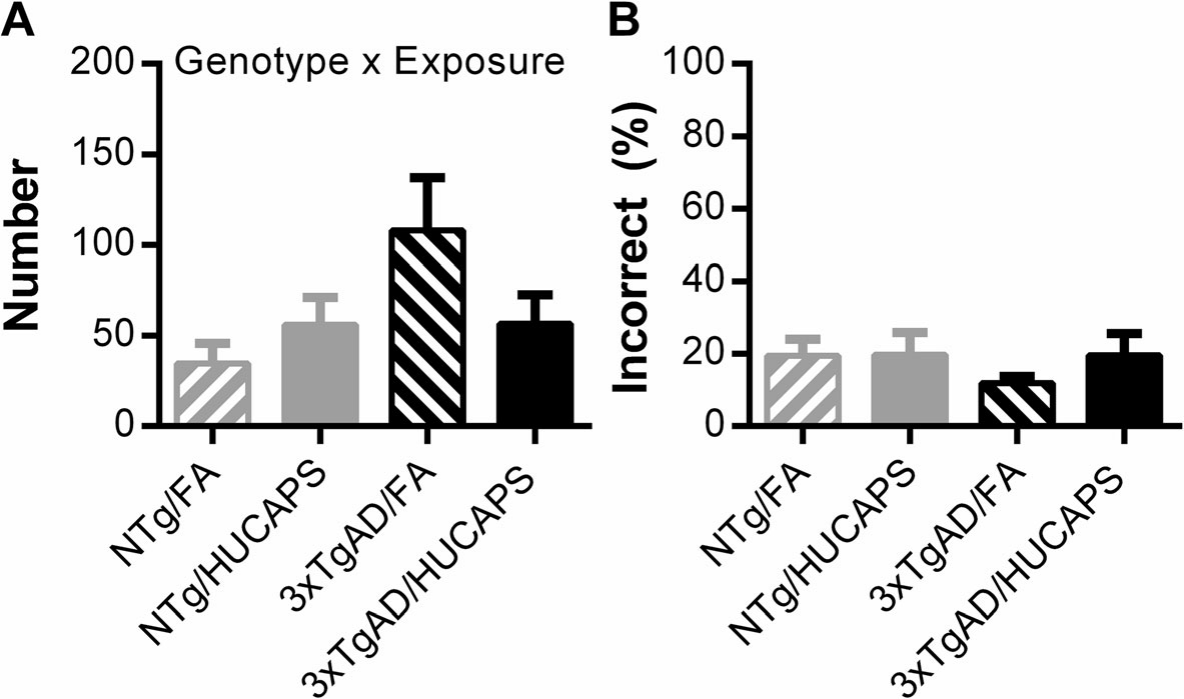
PR Schedule of Reinforcement: Measures of breakpoint (**a**) and percent inactive (incorrect) lever responses (**b**). Post-hoc Tukey-Kramer group comparisons were performed on breakpoint. Bars indicate the group means ± SE. Only significant results are reported at top of the figure. *n* = 8–11 per group

Appetitive motivation, assessed using a PR schedule of reinforcement, was pursued due to the dependence of RAM on food motivation. Statistical analysis of the breakpoint measure (final ratio size) of the PR schedule showed no significant main effect of genotype or HUCAPS exposure. However, a significant interaction between genotype and exposure (β = − 0.344, SE = 0.151, *p* = 0.030) on breakpoint was found, even when subject level change from ad libitum weight was included as a covariate in the model (Fig. 7a). Including the covariate term (β = 0.065, SE = 0.025, *p* = 0.013) in the model accounted for variation in weight loss, as this may be a potential confounding factor if otherwise overlooked. Post-hoc analysis of breakpoint, using Tukey-Kramer test, found no significant group differences in motivation.

There were no significant main effects or interactions on percent inactive (incorrect) lever responses among groups, suggesting that the changes seen in breakpoint (motivation measure) were not driven by inappropriate responding or differences in training (Fig. 7b).

### Breakpoint tested within RAM models

Considering that there appeared to be variations in motivation (interaction between genotype and exposure) that may have influenced outcomes in food motivated behavioral paradigms, like the RAM, statistical analyses tested whether individual-level differences in motivation could account for the effects observed in the RAM. This was tested by including the breakpoint value as a covariate in the original models used, and excluding subjects without PR data. The inclusion of breakpoint in the percent accuracy model did not improve the Akaike information criterion (AIC) value (original AIC: 19666.5, new AIC: 19666.7), an estimator for the goodness of fit of a model. The lack of model improvement with breakpoint inclusion was also noted in the analysis of both working memory phases: acquisition (original AIC: 1864.8, new AIC: 1865.4) and performance (original AIC: 6244.2, new AIC: 6244.5). Analysis of the acquisition phase of reference memory showed a slight improvement of model fit with the inclusion of breakpoint data (original AIC: 2581.4, new AIC: 2580.5) where the breakpoint term approached significance (β = − 0.006, SE = 0.004, *p* = 0.084). Analysis of the performance phase of reference memory did not show an improvement of model fit with the inclusion of breakpoint data (original AIC: 7057.1, new AIC: 7057.6). This indicates that variations in motivation may have influenced the acquisition, but not the performance, of reference memory and do not compellingly account for the observed changes in spatial learning which are primarily driven by performance phase alterations.

### Olfactory discrimination testing

**Fig. 8:**
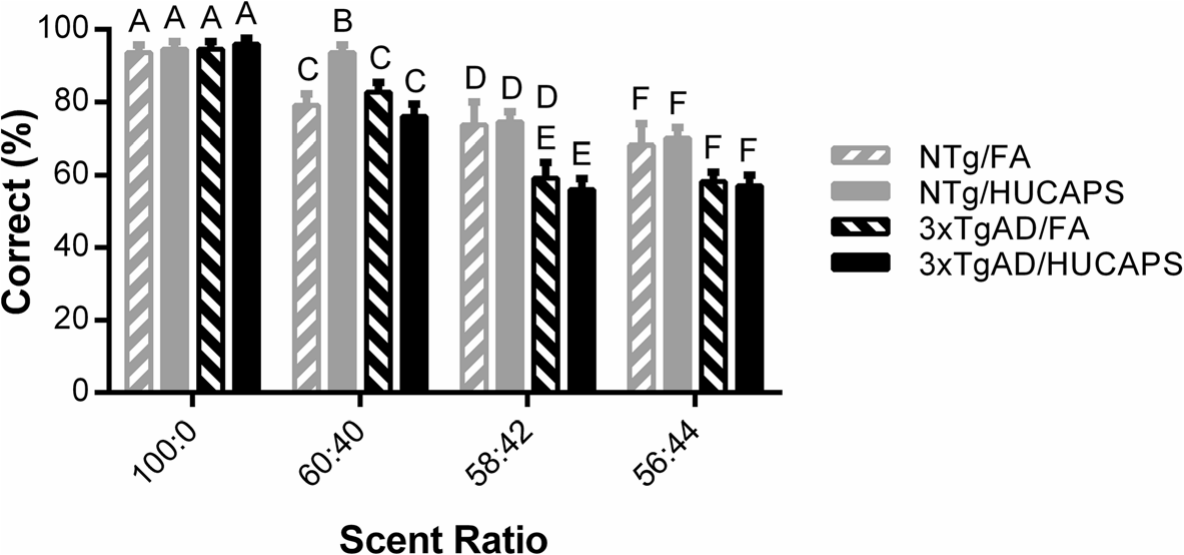
Olfactory Discrimination Testing: Percent correct choice for tested ratios 100:0, 60:40, 58:42, and 56:44. Post-hoc Tukey-Kramer group comparisons were performed within each ratio. Different letters denote significantly different groups. Bars show means ± SE. *n* = 10–11 per group

Main effects of discrimination ratio (*p* < 0.001) and genotype were found on percent correct choices in olfactory discrimination testing, with 3xTgAD mice showing lower discrimination accuracy than NTg (β = − 4.23, SE = 0.856, *p* < 0.001) (Fig. 8). Though no significant main effect of exposure was found, a significant genotype x exposure interaction (β = − 1.73, SE = 0.856, *p* = 0.045) and genotype x odor ratio interaction (*p* = 0.003) were present in olfactory discrimination testing. Odor ratios were independently explored via post-hoc Tukey-Kramer tests. At the 100:0 ratio (pure vanilla- or almond-scented water), no significant differences between groups were found, indicating a similar level of odor discrimination. At the more difficult discrimination ratio of 60:40, post-hoc analysis showed that the NTg HUCAPS-exposed mice had better olfactory performance (higher percent correct choices) as compared to the other groups. At the 58:42 ratio, 3xTgAD HUCAPS mice had the poorest olfactory discrimination performance of the four groups. No differences between the groups were found at the 56:44 ratio.

## Discussion

The present study assessed whether exposure to UFPs could enhance AD-related learning and memory deficits. We found that exposure to ambient UFPs in aged male mice resulted in protracted detriments in spatial learning, reference memory, and short-term memory (Table 2). Interestingly, these alterations were independent of underlying AD genotype, suggesting that the changes caused were independent of pre-existing AD pathology or cognitive deficits. Whether these alterations were due to the promotion of AD-related pathology is not clear, as we did not explore tissue endpoints in this study. However, these findings support the reports of an increased prevalence of MCI (the cognitive transitional state between normal cognitive aging and potential dementia) in association with higher lifetime PM_2.5_ exposures [39]. The findings are notable given that 91% of the world’s population live in areas where PM_2.5_ concentrations, of which UFPs are a significant proportion, exceed annual mean World Health Organization air guidelines. This raises the possibility that millions of people are potentially at risk for cognitive decrements [40] and/or elevated AD risk [11] in response to air pollution exposure.

**Table 2:** Summary of behavioral testing results

Behavioral measure	Results
Locomotor Activity Testing	No change
RAM: Spatial Learning	HUCAPS ↓ learning 3xTgAD ↓ learning rate
RAM: Working Memory	3xTgAD ↑ working memory error rate
RAM: Reference Memory	HUCAPS ↑ reference memory errors
NOR: Session 2	HUCAPS & 3xTgAD impaired short-term memory
Progressive Ratio Schedule	HUCAPS altered motivation in genotype-dependent manner
Olfactory discrimination	Ratio-dependent 3xTgAD deficits in olfactory discrimination HUCAPS improved olfactory discrimination in NTg and worsened discrimination in 3xTgAD mice

The 3xTgAD mouse model recapitulates many aspects of the early stages of human AD, including early cortical and medial temporal lobe (MTL) pathology [41–43]. Clinical neuropsychological assessments for AD include tests that probe the function of affected neuroanatomical structures, including those for both semantic (reference) and working memory [44]. We used rodent behavioral assays to test analogous cognitive domains, particularly probing MTL/hippocampal function through spatial learning and memory and short-term recognition memory. Reference memory, working memory, and recognition memory are all supported, at least at some phase of the learning/recall process, by MTL function in both humans and rodents [45–47]. Cognitive changes in male 3xTgAD mice are generally described as initial deficits in spatial working memory at ~ 4–6 months of age, followed by deficits in short-term recognition memory at ~ 9–11 months [37, 44, 48], and finally, by measurable impairments in reference memory. However, the appearance of these latter deficits are more variably-described in the literature as starting from 2 to 9 months [36, 37] or not at all up to 12-months of age [49].

Our study was limited to male mice because the timeline of AD pathology and behavioral changes in the 3xTgAD mouse model is better characterized in males than in females. Further studies are required to understand potential sex-specific effects of UFPs on AD-related cognitive changes. This is especially important, as AD is thought to be more prevalent in women, with some studies finding women to have an almost two-fold increased risk of developing AD after the age of 65 compared to men of the same age [50]; however, others do not find sex-specific differences in AD risk [51]. Conversely, there is a growing body of research indicating that air pollution exposure is worse for men’s cognitive health [52, 53] adding further to the uncertainty of how sex specific differences may influence cognitive alterations following air pollution exposure.

### Exposure

Interestingly, the UFP effects seen in our study occurred after a short-duration exposure (~ 2-weeks), which was implemented to explore if an episodic paradigm could result in detectable behavioral alterations, as has been shown in neonates that were exposed to HUCAPS aerosols using a similar paradigm [54, 55]. Our results suggest that even brief exposures to UFPs in adulthood can have negative impacts that, when considering the potential cumulative impacts of a lifetime of exposure, may be of significant concern [52]. A limitation of this study is that it was not designed to interrogate the immediate effects of HUCAPS exposure on lung or systemic inflammation or neuropathological changes; however, we do not typically find any indication of extant pulmonary inflammation immediately following HUCAPS exposure (data not shown).

We utilized the HUCAPS to selectively concentrate UFPs from roadside ambient air, providing a real-world mixture of PM for exposure. Because the concentrator relies on ambient UFP concentrations, the temporal variations during HUCAPS exposure in number concentration, and presumably particles size variability, closely parallel the variability in outdoor PM characteristics (Additional file 1: Supplemental 6). We do not yet know if compositional variability contributes to the response. However, the day with the highest exposure number concentration (Day 4) was the calmest in that there were three consecutive hourly readings of wind speeds at 0 mph, recorded at a nearby weather station, with winds coming out of the north and no precipitation, conditions that we have noted to be associated with higher number concentrations in outdoor and, therefore, concentrated air. Nevertheless, the use of a concentrated ambient mixture offers advantages over the use of laboratory-generated aerosols that may not accurately reflect real-world exposures. In terms of the relevance of the UFP aerosol concentrations, the HUCAPS number concentration (122,000 particles/cm^3^) is well within what a person might experience along a highly-trafficked roadway (Fig. 1). Particle number concentrations have been measured to be 150,000 to 400,000 particles/cm^3^ near highways like those in Minneapolis, MN and Los Angeles, CA [56, 57].

Dosimetry modeling was used to provide some context about how the daily deposited dose following HUCAPS exposure might compare to estimated human UFP exposures under real-world conditions. A study that modeled human ambient UFP exposures in Manila, Philippines, estimated daily deposited doses in the range of ~ 5–34 μg [34]. Scaling for species differences in respiratory tract surface area, we estimate that the average HUCAPS deposited dose in the mice was ~ 0.4 ng/cm^2^, whereas it would range from ~ 0.006–0.04 ng/cm^2^ in humans based on the example above (Additional file 1: Supplemental 7).

### Radial arm maze

The conversion rate of MCI to AD is around 7% [58]. Early impairments in semantic memory (knowledge of concepts) and working memory (manipulation of short-term memory) have been demonstrated to be strong predictors of progression from MCI to AD [4] and are predominantly affected in prodromal and mild AD stages, typically preceding decline in other cognitive domains [59, 60]. Both semantic memory (analogous to reference memory in rodents [7]) and working memory can be assessed using the RAM, making it a strong behavioral paradigm to test AD- and MCI-related alterations. Reference memory, memory consistent across sessions (i.e., location of rewarded arms), and working memory, memory used within a single session (i.e., what arms were already visited) are components that are crucial for the formation and maintenance of spatial information.

RAM testing was not confounded by differences in locomotor activity as determined by SLA testing (Fig. 2) and mean time per arm in RAM testing (Fig. 3c). The predominant change in learning phenotype observed in RAM testing was in transgenic mice, where 3xTgAD mice had diminished spatial learning resulting from working memory deficits compared to NTg mice (Fig. 3a), which is expected for 3xTgAD mice at this age. These changes were observed on the rate (slope) component of the performance phase (Fig. 5a) and approached significance in the assessment of mean performance phase errors (Fig. 5b). Our findings, that AD background selectively impaired working memory while leaving reference memory unaltered, are consistent with the RAM findings of Gabbita et al. [61], which tested 6-month old male 3xTgAD mice. The majority of studies in 3xTgAD mice report age- and pathology-dependent effects on spatial learning, with deficits typically developing at ~ 6 months of age, although the Morris water maze test is more frequently used [37, 62, 63].

HUCAPS exposure reduced mean percent accuracy across approximately the last third of the test sessions (Fig. 3b); these reductions were actually present in the mean percent accuracy from all sessions (Additional file 1: Supplemental 8), indicating a persistent deficit. This appeared to arise from reference memory alterations (Fig. 4), which likewise appeared to be persistent (Fig. 4b) (Additional file 1: Supplemental 3). These findings are consistent with other studies of adult animals that were exposed to PM. Four week-old C57BL/6 mice exposed for 10 months to concentrated PM_2.5_ showed impaired spatial learning and reference memory in the Barnes maze, specifically an increased latency to reach the target hole over 4 days of testing [64]. Another study showed that spatial learning deficits in Morris water maze performance occurred in a dose-dependent manner in 6 week-old BALB/c mice exposed for 3 months to nanoparticle-rich diesel exhaust aerosols [65]. The absence of HUCAPS-induced working memory deficits is consistent with other PM exposure studies performed in 10 week, 3 month, and 18 month-old mice exposed for 3–13 weeks followed by spontaneous alternation behavior assessment, which probes spatial working memory [66, 67].

While an interactive effect of HUCAPS by transgene was proposed in our hypothesis, it is also evident that the ability to detect such effects may be limited by a floor effect in RAM performance given that mean percent accuracy of the AD HUCAPS group, tested by a one sample t-test to 50% (e.g. clockwise or counter clockwise search strategy), was at chance levels (Fig. 3b). This means further reductions were unlikely barring bizarre behavioral patterns such as stereotypy.

The delayed manifestation of an AD-driven effect suggests a temporal and regional component to the development of the pathology underlying the appearance of working memory deficits. HUCAPS-induced reference memory alterations, which were detected in the performance phase, may reflect memory retrieval deficits. Where working memory is applicable to an individual session, reference memory is utilized across all sessions of testing. Therefore, deficits in the ability to retrieve stored spatial information would greatly influence reference memory ability in the performance phase. Hippocampal lesion experiments have shown that the hippocampus is important in initial learning and consolidation of both working and reference memory, but hippocampal ablation after a task is learned results in minimal effects on reference memory, with recall involving cortical areas [68], but protracted deficits in working memory function [47, 69], providing some hint as to the potential mechanisms underlying the effects we observed.

### Novel object recognition testing

NOR performance was not influenced by exposure or genotype as tested by 2-factor analysis of variance (ANOVA) (Fig. 6b). Under these testing conditions, as compared to fictive data sets generated from a 50% population mean and with its own standard deviation, only the NTg FA-exposed group significantly preferred the novel object (Additional file 1: Supplemental 5), again highlighting the floor effect limiting our ability to detect changes by 2-factor ANOVA. The fictive dataset comparison statistical approach, utilized to determine preference for novel above chance, is more stringent than other traditional methods that may inflate type 1 error rate [70]. Utilizing comparisons to multiple fictive datasets and determining convergence of conclusions (i.e., all comparisons significant), allows more confidence in the conclusions and accounts for the variability in fictive data set generation. However, there may also be issues with respect to the context from which such data sets are generated (i.e., what is the true population standard deviation, testing conditions and parameters, and prior behavioral history of the mice).

The fact that neither session 1 outcome measures differed by conditions (Fig. 6a) nor was there evidence of neophobia (Additional file 1: Supplemental 9) suggests that the lack of preference for the novel object could be due to short-term memory deficits in the HUCAPS-exposed groups and 3xTgAD mice. The short-term recognition memory dysfunction in 3xTgAD mice that was found in this study at ~ 17-months is consistent with results observed in other studies of NOR, one reporting deficits as early as 3-months, while another describes deficits beginning at ~ 9-months of age [71, 72]. The literature on the relationship between PM exposure and NOR testing suggests a dose-dependent detriment in short-term recognition memory. Win-Shwe et al. [73], utilizing freshly generated nanoparticle-rich diesel engine exhaust, found effects on NOR at 4.39 × 10^6^ particles/cm^3^ following 3-months of exposure, but not after at 2.21 × 10^6^ particles/cm^3^. Interestingly, we observed alterations in novel preference after a lower concentration and shorter duration exposure than performed in that study. Differences in findings may result from differences in PM origins, composition and size, statistical methods utilized, and age of the mice at exposure.

### Progressive ratio and impact on radial arm testing

HUCAPS exposure was found to alter motivation in a genotype-dependent manner as assessed using a PR schedule of reward, an assay yet to be described for the 3xTgAD model (Fig. 7). Given that the RAM task is appetitively-motivated and, therefore, degree of food motivation could potentially confound RAM results, the PR breakpoint was tested as a covariate within the RAM models to determine the amount of variation in behavior that could potentially be attributed to differences in motivation. Assessment of RAM reference memory errors indicated that once the task was learned, reference memory was minimally influenced by motivational differences. All other measures from RAM testing, including spatial learning and working memory, showed no significant influence of motivation on variation. These results, in conjunction with our findings that HUCAPS exposure or genotype effects were detectible primarily in the performance phase, suggest that our outcomes were not significantly influenced by motivational differences.

### Olfactory discrimination testing

3xTgAD mice had diminished olfactory sensitivity compared to NTg mice (Fig. 8), findings that are consistent with observations in human AD and in other studies characterizing olfactory deficits in the 3xTgAD model [6, 74]. It is important to consider if this deficit may potentially underlie the working memory alterations observed in RAM testing, as olfaction in rodents is important for navigation [75]. It is difficult, if not impossible, to determine whether the measured genotype effect in working memory resulted from an actual deficit in working memory or was due to a diminished navigational ability in 3xTgAD mice caused by impaired olfaction. Scent cues left by a mouse in a visited arm would help the mouse distinguish which arms they already entered. An inability to utilize these scent cues might lead to elevations in working memory errors. However, it is unclear whether olfactory deficits were actually present during RAM testing, as olfactory testing and RAM testing were separated by approximately 4 months.

HUCAPS exposure altered olfaction, appearing to enhance olfactory discrimination in the NTg mice only, best illustrated at the 60:40 ratio. This is an unexpected finding, especially as human epidemiologic studies suggest that air pollution exposure has a negative impact on olfaction [76]. The results indicate that HUCAPS exposure may be acting on the olfactory pathway to induce a compensatory effect in the NTg mice. Interestingly, the general pattern of olfactory modulation (HUCAPS increasing response in the NTg, but decreasing response in 3xTgAD mice) is mimicked in the PR measure. Both olfaction and motivation are functionally interconnected through the medial olfactory tubercle (mOT), a ventral tegmental area dopaminergic pathway which has been shown to influence both reward and olfactory behavior [77]. The mOT shows evidence of sensitivity to oxidative stress following diesel exhaust exposure, which may result from the particular vulnerability of dopaminergic neurons or the fact that the mOT receives direct neural projections from the olfactory bulb, potentially providing a direct route of action for UFPs [78–80]. This proposed mechanism of action on these systems is purely speculative and will need further experiments to verify.

## Conclusion

Major findings from this study showed that short-term exposures to concentrated ambient UFPs at human-relevant concentrations resulted in protracted behavioral alterations in aged male mice (< 13 mo), impairing spatial learning through deficits in the reference memory domain, independent of AD mouse background and in the absence of locomotor and appetitive motivational influences. In contrast, AD-related spatial learning detriments appeared to involve working memory deficits, potentially resulting from diminished olfactory discrimination capability. This suggests that UFP and AD-related spatial learning deficits operate through distinct memory domains that converge on spatial memory. Both AD background and UFP exposure impaired novelty preference, indicating both AD status and HUCAPS exposure resulted in deficits in short-term recognition memory. Detection of additive or synergistic detriments in HUCAPS exposed 3xTgAD mice on these cognitive domains was not observed, but may have been hindered by intrinsic testing limitations (floor effect). This study provides further context to the ongoing discussion regarding how air pollution exposure may be influencing cognitive health and AD-related risk. Future studies will need to address potential sex specific susceptibility to UFP-induced cognitive effects and whether exposure to UFP air pollution may enhance the onset of cognitive deficits in relation to AD initiation and the underlying alterations that give rise to these cognitive changes.

## Methods

### Animal model

3xTgAD and NTg mice were the progeny of founder mice gifted to Dr. William J. Bowers (formerly of the University of Rochester) from Dr. Frank LaFerla (UC Irvine). The 3xTgAD mice express amyloid precursor protein (APP)_Swe_, tau_p301L_, and presenilin 1 (PSEN1)_M146V_, resulting in the development of extracellular Aβ plaques by 6 months of age and tau tangle pathology by 12 months of age [35, 81]. Our lab has confirmed the presence of both Aβ plaques and phosphorylated tau pathology in male 3xTgAD mice at ~ 12.5 months of age via 6E10 and pT205 staining respectively (Additional file 1: Supplemental 10). Mice were housed in rooms with a 12-h light/dark cycle with ad libitum access to food and water in an Association for the Assessment and Accreditation of Laboratory Animal Care International (AAALAC) accredited vivarium. Two weeks prior to exposure, mice were given tail tattoos for identification purposes. All animal exposures and behavioral tests were approved by the University Committee on Animal Resources.

### Exposure

A total of 21 3xTgAD and 22 NTg male mice underwent whole-body inhalation exposures with *n* = 10–11 of each genotype undergoing either UFP or high-efficiency particulate air (HEPA)-filtered air exposure. The inhalation chambers had partitions so that each mouse was kept separate during the duration of exposure. Only male mice were used for the study due to limited capacity in the whole-body inhalation chambers and the timeline of pathology is better characterized for the male 3xTgAD mice than for the females of this strain. The mice were approximately 12.5 months old at the beginning of exposure. Exposures took place for 4 h per day, 4 days per week, for 2 weeks (5/9–5/18/17; 10 am – 2 pm). Body weights were collected periodically across the course of exposure. Body weights dropped initially at the beginning of exposure but then remained consistent across exposure days (Additional file 1: Supplemental 11).

The use of HUCAPS provided a real-world UFP mixture for exposure, where UFPs were concentrated ~ 5× that of ambient air number concentration; the gaseous components were neither eliminated nor concentrated in the system. Ambient near-roadway air in Rochester, NY was drawn into the HUCAPS at a rate of 5000 L/min through a size-selective inlet, where a series of inertial cascade impactors removed particles that were larger than 2.5 μm from the aerosol. The aerosol then underwent condensational growth followed by virtual impaction to selectively concentrate ultrafine particulates. The aerosol was dehydrated and passed through a final size-selective outlet that removed particles larger than 0.2 μm just before release into the exposure chamber [82].

Both the efficiency of the concentrator and therefore the concentration of the aerosol, along with its composition, are dependent on ambient conditions, resulting in temporal variability in concentration and composition. The aerosol number concentration and size distribution were monitored in the exposure chamber by a scanning mobility particle sizer composed of a differential mobility analyzer and a condensation particle counter (model 3071 and model 3022 A, respectively; TSI, Shoreview, MN). Mass concentrations were determined each exposure day via gravimetric sampling. The fractional regional deposition in the respiratory tract was determined with the MPPD model (v 3.04) using allometrically scaled respiratory rate and tidal volume values [38, 83, 84]. The estimated daily deposited dose was then calculated for each region assuming constant average mass concentration and UFP size distribution (GSD, CMD), and allometrically scaled minute ventilation (Additional file 1: Supplemental 12) [85].

### Overview of behavioral testing

Following exposure, mice were calorically restricted to approximately 85% of their ad libitum weight to enhance motivation and reinforcement prior to the beginning of behavioral testing and were maintained at this weight for the duration of testing. Subsequent olfactory discrimination testing was conducted using water restriction, which was initiated after the mice were returned to an ad libitum diet. There was no significant difference between NTg (34.22 g ± 4.49) and 3xTgAD (36.12 g ± 3.99) ad libitum body weights measured prior to exposure (Additional file 1: Supplemental 13). Behavioral testing began one-month post-exposure after target caloric restriction body weights were attained. Behavioral testing was performed in the sequence described in Table 1 during the light phase of the light-dark cycle unless otherwise noted. All testing apparatuses were thoroughly cleaned with disinfectant between animals.

### Spontaneous locomotor activity

Automated locomotor activity chambers were used (Opto- Varimex Minor, Columbus Instruments, Columbus, OH) to assess SLA. The chambers used are transparent acrylic arenas equipped with three planes of infrared photobeams that detected activity in the x-, y-, and z-planes. The SLA arenas were housed in sound attenuating chambers to minimize distractions. A single 60-min session was performed and measures were aggregated into 5-min bins. A 2 × 2 photobeam box is defined within the program’s parameters from which various activities can be categorized. Ambulatory counts (only parameter reported here) were defined as successive breaks of the 2 × 2 photobeam box as the mouse moves about the chamber.

### Radial arm maze

Hippocampal dependent learning and memory was assessed using a standard polypropylene 8-armed RAM (Med Associates, MED-RAMMN, Fairfax, VT). The maze consisted of 8 equally spaced arms radiating from an octagonal central platform. The walls of the maze were clear, allowing the use of extra-maze visual cues for spatial orientation. Mice were habituated to the maze in 3 trials where mealworm rewards were scattered in the maze and the mouse allowed to freely explore. Across the 3 habituation trials, the reward placement was gradually restricted to the arm ends where the rewards would be located during testing. Habituation trials ended once all rewards were collected. Mealworms were used as we have found these to be highly preferred, readily consumed, and less disruptive of normal nutritional intake (a potential source of confounding) compared to other potential rewards (e.g. glucose pellets, cookies, etc.) [86].

For testing, 4 different baiting schemes were created where 4 of the 8 arms would hold reward and no more than 2 adjacent arms were rewarded. A baiting scheme was assigned to each mouse and used throughout testing. A mouse was placed in the center of the maze and allowed to enter the arms freely. Number of correct entries into baited arms, working memory errors (entries into previously baited arms from which reward had already been collected) and reference memory errors (entries into any arm that never held reward) were measured within each session. The session ended after all 4 rewards were collected or after a maximum session time of 20 min elapsed, whichever occurred first. The mice were tested in single daily sessions for a total of 64 sessions. The maze was thoroughly cleaned with disinfectant between tests of individual mice to prevent carryover of scent cues. Percent accuracy was calculated as an assessment of spatial learning [percent accuracy = number of correct entries/total number of arm entries × 100].

### Novel object recognition

In session 1, each mouse was placed in a 30.5 cm × 30.5 cm × 30.5 cm Plexiglas arena with two identical white, porcelain doorknobs for 30 min. The mouse was then returned to its home-cage for 24 h, after which session 2 took place. In session 2, the mouse was placed back into the arena for 10 min now with one of the familiar white door knobs from session 1 replaced with a grey metal doorknob (unfamiliar object). Placement of the novel object in the arena was counterbalanced to avoid potential side bias.

All sessions were video recorded for behavior scoring. The reviewer scored the number of interaction bouts and the duration of interactions with either object for the total 30 min of session 1 and the first 6 min of session 2. Interactions were counted as the entry of the nose or head into the object zone (~ 2 cm around object). From session 2, the recognition time index was calculated as follows [RI (time) = time with novel/total interaction time × 100].

### Progressive ratio testing

This behavioral paradigm was conducted in operant chambers (Med Associates, St. Albans, VT) housed in sound attenuating and ventilated cabinets during overnight sessions. In the chamber, three levers were located along the wall opposite of the pellet dispenser. Mice were trained to press an assigned lever for food reward reinforcers (20 mg food pellet) using a variable time 60 s fixed ratio 1 schedule (VT60FR1). In this schedule, a reinforcer was delivered simultaneously with a light and sound cue every 60 s independently of behavior. Any responses on the designated correct lever during the session would also trigger the light and sound cue along with reinforcement delivery. After 20 min on the VT60FR1 schedule, only responses on the designated correct lever produced food reward. The mouse was considered trained once it collected at least 35 lever-associated reinforcers in a single overnight session. 

Once trained, a PR schedule was implemented to evaluate food-related motivation. The PR schedule requires a fixed number of responses on the correct lever for each reinforcer delivery. The ratio of responses for each reward increases after each reward delivery following a protocol described in Cory-Slechta et al. [55]. The session ended when 5 min elapsed without any responses. The ratio at which the session ended is defined as the breakpoint or the limit to which the amount of effort required outweighs the benefit or perceived value of the food reinforcer. The progressive ratio program shut off prematurely for six animals, so their data were excluded from the analysis. This reduced the sample size to *n* = 8 for the 3xTgAD FA and 3xTgAD HUCAPS groups.

### Olfactory discrimination testing

A protocol to test the ability of mice to discriminate between scent mixtures (100:0, 60:40, 58:42, 56:44) was adapted from Enwere et al. [87]. Mice were habituated to a 30.5 cm × 30.5 cm × 30.5 cm Plexiglas arena in which training and testing phases were performed. Mice were water restricted for 32 h prior to training and testing. Just prior to training and testing sessions, the mouse was presented with 20 μl of plain water in a 35 mm × 10 mm tissue culture dish to assess thirst. The mouse was allowed a maximum of 4 min to approach and drink from the dish, after which both the mouse and dish were removed.

In the training phase, each mouse underwent positive scent training followed by negative scent training in which the mouse was presented with a dish of 12 μl of water with 8 μl of either almond or vanilla extract. In the positive training phase, the water was plain and scented with extract (e.g., vanilla) [+], while in negative training the water was a 1% solution of denatonium benzoate that was scented with the opposite extract (e.g., almond) [−]. The assignment of the two extract mixes as positive or negative stimuli was counterbalanced across mice to prevent scent preference bias, but was held consistent for an individual mouse. In a positive scent training trial, a mouse was presented with the [+] dish randomly placed within the chamber. The trial ended when a maximum trial time of 4 min elapsed or the mouse finished the dish of water. The mouse and dish were removed at the end of the trial. Positive training was considered complete once the mouse drank from the [+] dish for 5 consecutive trials without exceeding each trial time limit. Negative scent training followed positive scent training utilizing a similar paradigm, but the mouse was considered trained when it refused to drink from the [−] dish for 5 consecutive trials.

In the testing phase, 100:0, 60:40, 58:42, 56:44 scent ratios were tested on separate days to avoid satiation. The mixtures consisted of both [+] and [−] associated scents with the dominant scent in the mixture meant to indicate whether the liquid would be either palatable (e.g. vanilla:almond at 60:40) or not (e.g. almond:vanilla at 60:40). Just prior to the start of each day’s testing, there were introductory trials where the scent mixture being tested that day was introduced one at a time with 2 presentations of the [+] dish followed by 2 presentations of the [−] dish. After the introductory trials, testing began. Each subject was given 10 testing trials where the [+] and [−] dish were randomly placed in the arena. The trial ended once the mouse drank from the [+] correct dish, drank from the [−] incorrect dish, or 4 min elapsed. The latter 2 outcomes were considered incorrect.

### RAM statistical analysis

All raw RAM data were analyzed using a linear mixed effects model approach using the “lme4” package (v.1.1–19) of R [88]. All measures from the RAM were analyzed independently. A random intercept and slope model was used to account for subject-level variability on average performance and learning rate respectively with data centered at the initial session. Slope components were used to define the linear learning function (rate) across the sessions. Session, genotype, and exposure were designated as fixed effects within the model. The AIC value was used to determine whether the interaction between genotype and exposure should be included within the model. The model with the lowest AIC value was selected. The results are presented as the parameter estimate (β), standard errors (SE), and significance tests. The reference groups from which the parameter estimates were compared were the NTg mice and/or FA exposure. The “lmerTest” R package was used to compute the reported *p*-values.

For some of the RAM measures, there was a clear asymptotic approach to the value 0 (i.e., working memory errors, reference memory errors). In these cases, a piecewise model approach was used in which the measures were separated into two phases: the beginning acquisition phase and the subsequent performance phase. Each phase was then analyzed separately as previously described for non-linear learning curves [89, 90]. The inflection point that divided the two phases was determined by graphical analysis of NTg FA performance for each error measure. For reference memory errors, the acquisition phase was sessions 1–13 and the performance phase was sessions 14–64. For working memory errors, the acquisition phase was sessions 1–11 and the performance phase was sessions 12–64. This split analysis allowed us better resolution of the changes across testing, as the acquisition phase had a much steeper slope than the performance phase and, thus, may have skewed or diminished the ability to detect nuanced changes in memory errors.

Mean percent accuracy of the last 25 sessions was tested by 2-factor ANOVA and by per group one-sided one-sample t-tests to determine whether the group’s terminal performance was significantly above chance (50%) (clockwise or counterclockwise search strategy). RAM models testing PR breakpoint data as a potential covariate excluded subjects without PR data. Models were then tested with and without breakpoint data (testing main effect term and session interaction) to determine model fit improvement via AIC comparison.

### Additional statistics

The following analyses were performed using JMP statistical software (v.13). Locomotor data was analyzed using repeated measures ANOVA. NOR session 1 data was analyzed using a 3-factor ANOVA with genotype, exposure, and object side as factors. NOR session 2 was analyzed by 2-factor ANOVA with genotype and exposure as factors, as well as one-sided t-tests comparing each group against 6 fictive groups (*n* = 11) generated from a hypothetical population with μ = 50, σ = 25.5 (σ is the value of the control NTg FA group SD). This was performed to determine if the groups preferred the novel object more than chance, 50%, a method described by Akkerman et al. [70], which provides a more stringent determination of novel preference than other traditional methods (see more in discussion). An experimental group was determined to show novel object preference if comparisons to all 6 fictive groups were significant (if all significant, only one of the *p*-values reported) (Additional file 1: Supplemental 5).

PR breakpoint data, which was log distributed, was transformed prior to analysis. Breakpoint data was then analyzed by 2-factor analysis of covariance (ANCOVA), with genotype and exposure as factors and the percent of original ad libitum bodyweight just prior to PR testing included as a covariate. Significant interactions in breakpoint data were explored using post-hoc Tukey-Kramer tests. Percent inactive lever responses were tested by 2-factor ANOVA, using genotype and exposure as factors. Olfactory discrimination testing was analyzed by 3-factor ANOVA with genotype, exposure, and scent ratio as factors. Individual ratios were then independently explored using post-hoc Tukey-Kramer tests. Outliers for each behavioral test were determined by the Grubbs’ test: one NTg FA mouse was excluded from SLA analysis. The a priori statistical significance criterion was α < 0.05. Text in the figures summarizes statistically significant findings.

## Additional file

**Additional file 1: Supplemental 1.** Radial arm maze percent accuracy. **Supplemental 2.** Radial arm maze reference memory errors. **Supplemental 3.** Reference memory errors, performance phase only. **Supplemental 4.** Radial arm maze working memory errors. **Supplemental 5.** Characteristics of randomly generated fictive data sets. **Supplemental 6.** Outdoor particle number concentration during HUCAPS exposure. **Supplemental 7.** Estimated surface area deposited doses in upper respiratory tract and alveolar regions. **Supplemental 8.** RAM percent accuracy across all 64 sessions. **Supplemental 9.** object recognition testing – Session 2. **Supplemental 10.** 6E10 and phospho-tau staining in hippocampus from ~12.5 month old NTg and 3xTgAD mice. **Supplemental 11.** Mouse body weight during exposure. **Supplemental 12.** MPPD inputs and scaling adjustments. **Supplemental 13.** body weight measured prior to exposure.

## Data Availability

The datasets used and/or analyzed during the current study are available from the corresponding author on reasonable request.

## Abbreviations

AD: Alzheimer’s disease
AIC: Akaike information criterion
ANCOVA: Analysis of covariance
ANOVA: Analysis of variance
APP: Amyloid precursor protein
Aβ: Amyloid-β
CMD: Count median diameter
CNS: Central nervous system
FA: Filtered air
GSD: Geometric standard deviation
HEPA: High-efficiency particulate air
HUCAPS: Harvard ultrafine concentrated ambient particle system
MCI: Mild cognitive impairment
mOT: medial olfactory tubercle
MPPD: Multiple-Path Particle Dosimetry
MTL: Medial temporal lobe
NOR: Novel object recognition
NTg: Non-transgenic
PM: Particulate matter
PR: Progressive ratio
PSEN1: Presenilin 1
RAM: Radial arm maze
RI (Time): Recognition time index
SD: Standard deviation
SE: Standard error
SLA: Spontaneous locomotor activity
UFP: Ultrafine particulate matter
