# Supplementary material for: Selective memory and behavioral alterations after ambient ultrafine particulate matter exposure in aged 3xTgAD Alzheimer’s disease mice
Source: Part Fibre Toxicol. 2019 Nov 26;16:45. doi: 10.1186/s12989-019-0323-3 (PMC6878709; doi:10.1186/s12989-019-0323-3)
Supplement: Supplementary file 1 — Additional file 1: Supplemental 1. Radial arm maze percent accuracy. Supplemental 2. Radial arm maze reference memory errors. Supplemental 3. Reference memory errors, performance phase only. Supplemental 4. Radial arm maze working memory errors. Supplemental 5. Characteristics of randomly generated fictive data sets. Supplemental 6. Outdoor particle number concentration during HUCAPS exposure. Supplemental 7. Estimated surface area deposited doses in upper respiratory tract and alveolar regions. Supplemental 8. RAM percent accuracy across all 64 sessions. Supplemental 9. object recognition testing – Session 2. Supplemental 10. 6E10 and phospho-tau staining in hippocampus from ~12.5 month old NTg and 3xTgAD mice. Supplemental 11. Mouse body weight during exposure. Supplemental 12. MPPD inputs and scaling adjustments. Supplemental 13. body weight measured prior to exposure. [file 12989_2019_323_MOESM1_ESM.docx]

**Selective memory and behavioral alterations after ambient ultrafine particulate matter exposure in aged 3xTgAD Alzheimer’s disease mice**

Katrina Jew, Denise Herr, Candace Wong, Andrea Kennell, Keith Morris-Schaffer, Günter Oberdörster, M. Kerry O’Banion, Deborah A. Cory-Slechta, and Alison Elder

**Additional File 1**

**Supplemental 1:** Radial arm maze percent accuracy


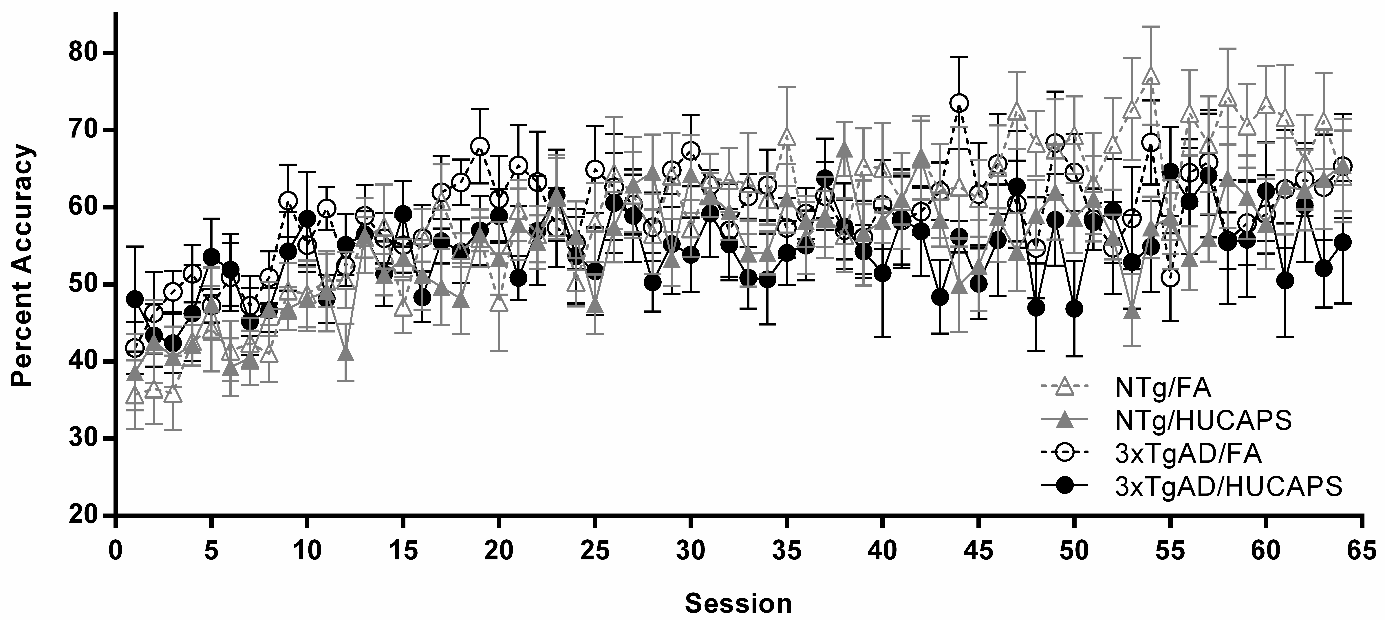


Percent accuracy was measured across 64 sessions of testing. Means ± SE. n = 10-11 per group.

**Supplemental 2:** Radial arm maze reference memory errors


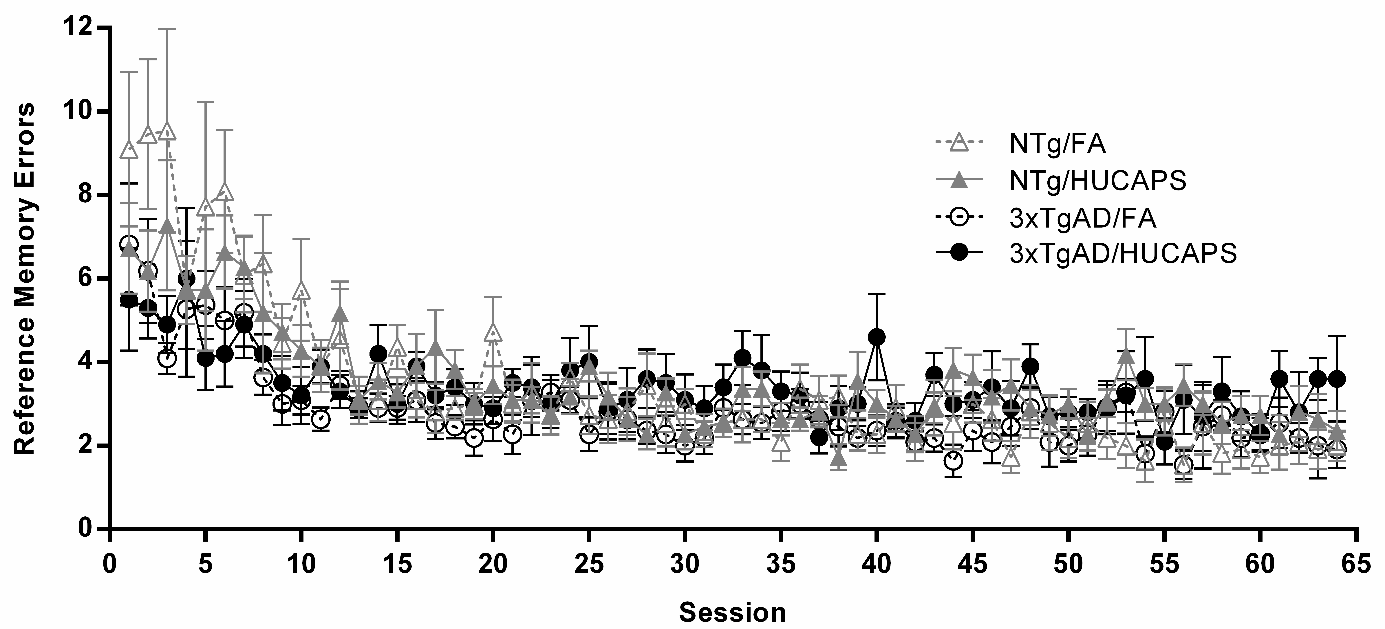


Reference memory errors were measured across 64 sessions of testing. Means ± SE. n = 10-11 per group.

**Supplemental 3:** Reference memory errors, performance phase only

**
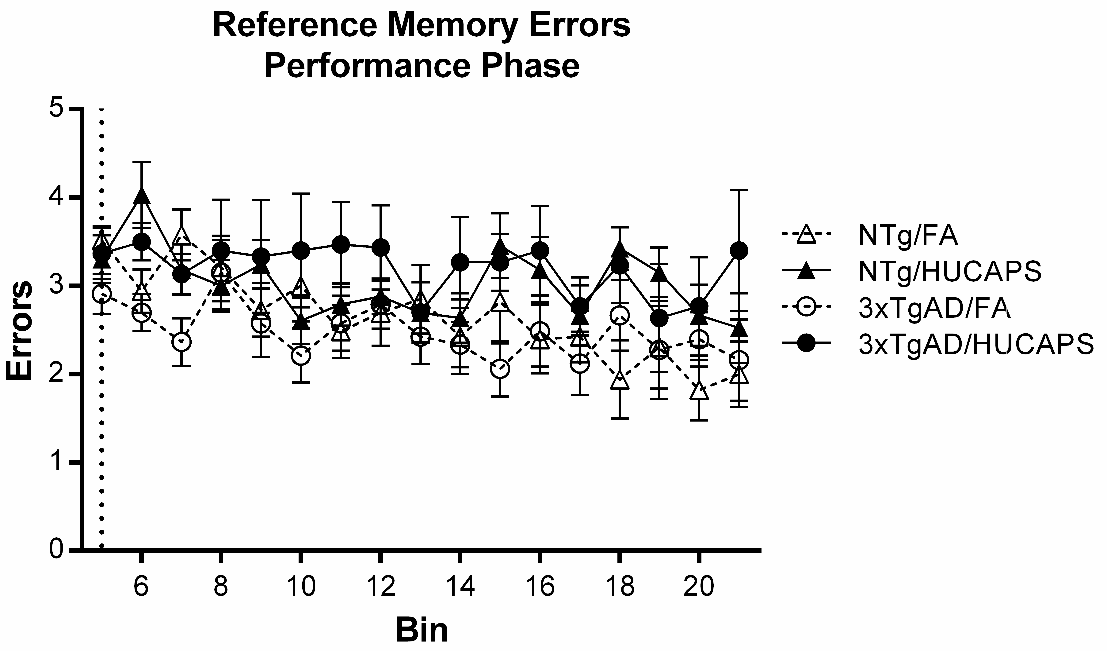
**

Approximate performance phase session division shown by dotted vertical line. Graph shows bins representing the mean errors from 3 consecutive sessions with the exception of bin 21, which is the average of 4. Means ± SE. n = 10-11 per group.

**Supplemental 4:** Radial arm maze working memory errors


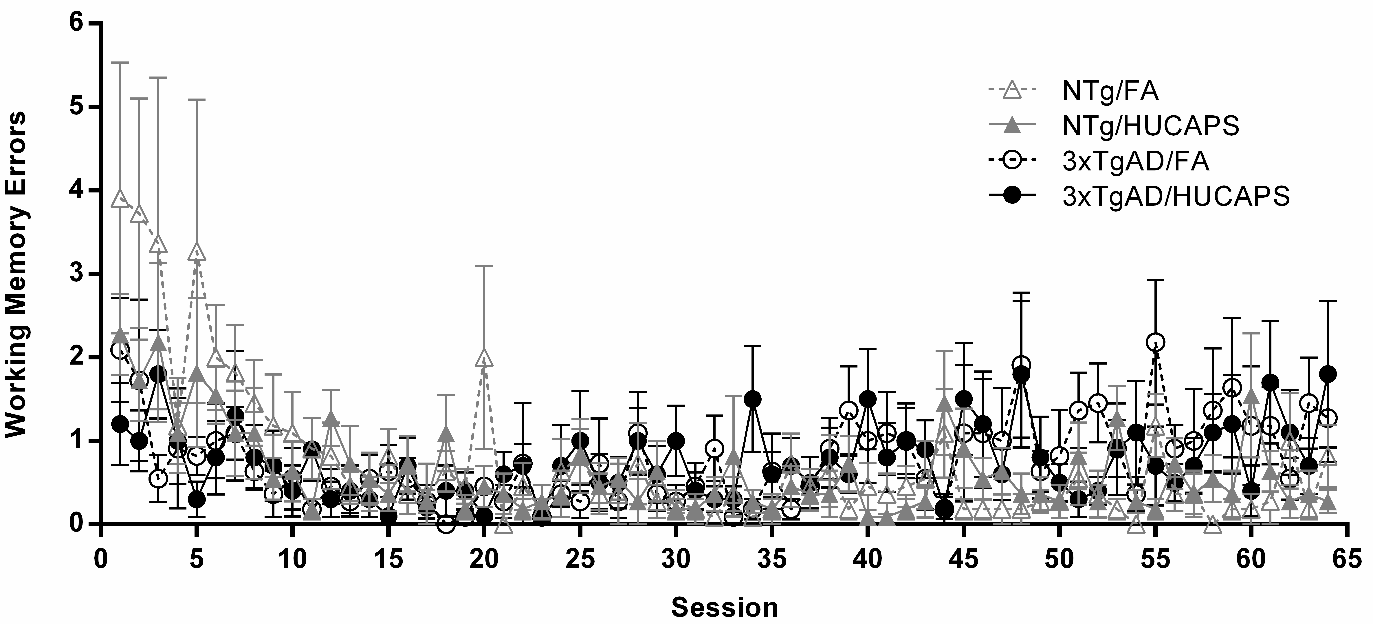


Working memory errors were measured across 64 sessions of testing. Means ± SE. n = 10-11 per group.

**Supplemental 5:** Characteristics of randomly generated fictive data sets

|  | **Fict. 1** | **Fict. 2** | **Fict. 3** | **Fict. 4** | **Fict. 5** | **Fict. 6** |
| --- | --- | --- | --- | --- | --- | --- |
| **Fict Mean** | 50.08 | 47.36 | 53.91 | 50.45 | 43.18 | 47.00 |
| **Fict SD** | 24.75 | 22.94 | 16.81 | 22.02 | 16.15 | 29.64 |
| **NTg**  **FA p>t** | p = 0.027 | p = 0.014 | p = 0.033 | p = 0.023 | p = 0.003 | p = 0.026 |
| **NTg HUCAPS p>t** | p = 0.184 | p = 0.115 | p = 0.264 | p = 0.180 | p = 0.035 | p = 0.142 |
| **3xTgAD FA**  **p>t** | p = 0.703 | p = 0.787 | p = 0.587 | p = 0.700 | p = 0.903 | p = 0.768 |
| **3xTgAD**  **HUCAPS**  **p>t** | p = 0.702 | p = 0.763 | p = 0.619 | p = 0.698 | p = 0.849 | p = 0.756 |

Data were (n = 11/data set) were generated from µ = 50, σ = 25.5 and the resulting p-values from one-sided t-tests of each fictive set to the experimental groups. Significant p-values are underlined.

**Supplemental 6:** Outdoor particle number concentration during HUCAPS exposure

| Day | Outdoor  Particle Number  Concentration Mean  (particles/cm^3^) | Outdoor  SD of Particle Number Conc. Mean |
| --- | --- | --- |
| 1 | 39819.0 | 29089.9 |
| 2 | 15691.7 | 12749.0 |
| 3 | 10932.5 | 8651.9 |
| 4 | 42962.5 | 9656.4 |
| 5 | 22476.8 | 12853.8 |
| 6 | 7464.0 | 6343.1 |
| 7 | 5294.1 | 1875.5 |
| 8 | 5095.2 | 1806.3 |
| Mean | 18717.0 | 10378.2 |
| SD | 15162.8 | 8680.3 |
|  |  |  |

Daily means and standard deviations (SD).

**Supplemental 7:** Estimated surface area deposited doses in upper respiratory tract and alveolar regions

| **Species - Measure** | **SA (cm^2^)** | **Estimated UFP Deposited dose (ng)** | **SA Deposited Dose (lung+URT) (ng/cm^2^)** |
| --- | --- | --- | --- |
| Mouse - URT SA | 2.89 [2] |  |  |
| Mouse - alveolar SA | 700 [3] |  |  |
| Mouse - URT + Alveolar SA | 702.89^*^ | 268^^^ | 0.4 |
| Human - URT SA | 196 [2] |  |  |
| Human - alveolar SA | 784,000 [4] |  |  |
| Human - URT + Alveolar SA | 784,196^*^ | 5,000-34,000 [1] | 0.006-0.04 |

Estimates for HUCAPS mouse exposures as compared to estimates from real-world exposures in humans [1].

(^*^) Surface area measures for tracheal-bronchial region not found so sum of URT and alveolar surface area (SA) used for calculation.

(**^^^**) Mouse estimated deposited dose from HUCAPS exposure and MPPD fractional deposition calculations.

**Supplemental 8:** RAM percent accuracy across all 64 sessions

**
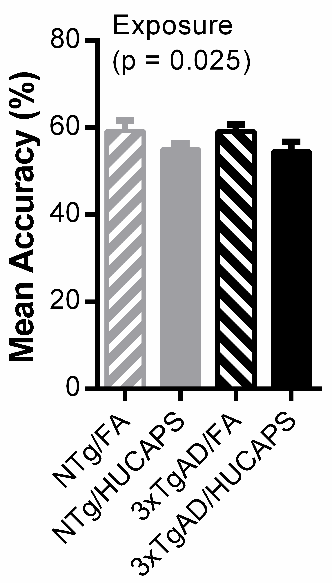
**

Means ± SE. Only significant results are reported at the top of the figure. n = 10-11 per group.

**Supplemental 9:** Novel object recognition testing – Session 2

**

**

Data depicting the number of interactions (bouts) towards novel and non-novel objects. Means ± SE.

**Supplemental 10:** 6E10 and phospho-tau staining in hippocampus from ~12.5 month old NTg and 3xTgAD mice

**
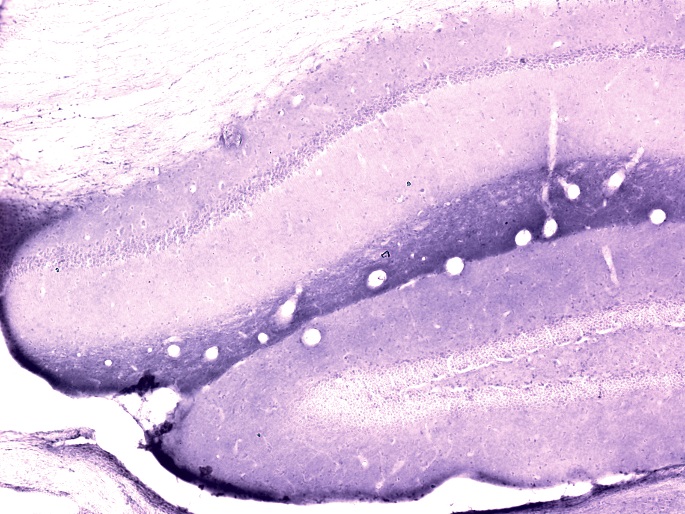

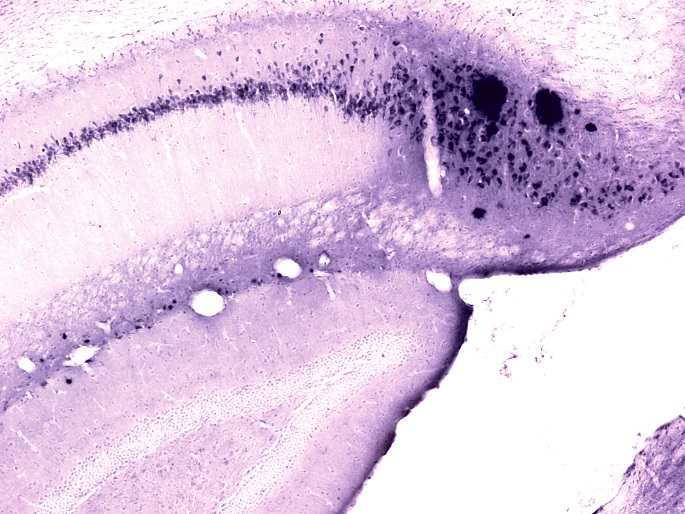
**

6E10 antibody staining of APP and Aβ in the hippocampus of ~12.5 month old NTg (Top - L) and 3xTgAD (Top-R) mice. Plaque pathology clearly visible in 3xTgAD mouse (100x magnification).

Tau phosphorylation staining using pT205 antibody in the hippocampus of ~12.5 month old NTg (Bottom – L) and 3xTgAD (Bottom – R) mice (40x magnification).


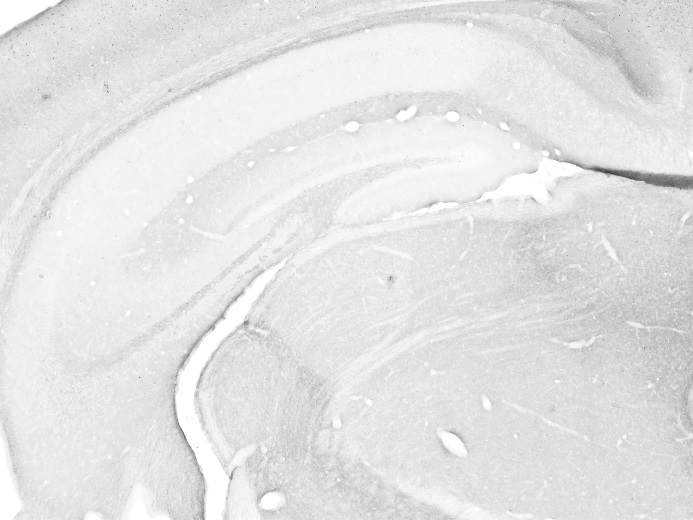

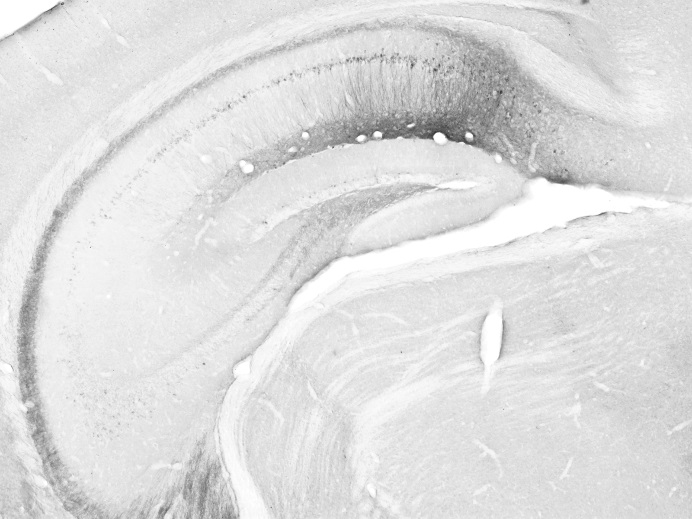


**Supplemental 11:**  Mouse body weight during exposure


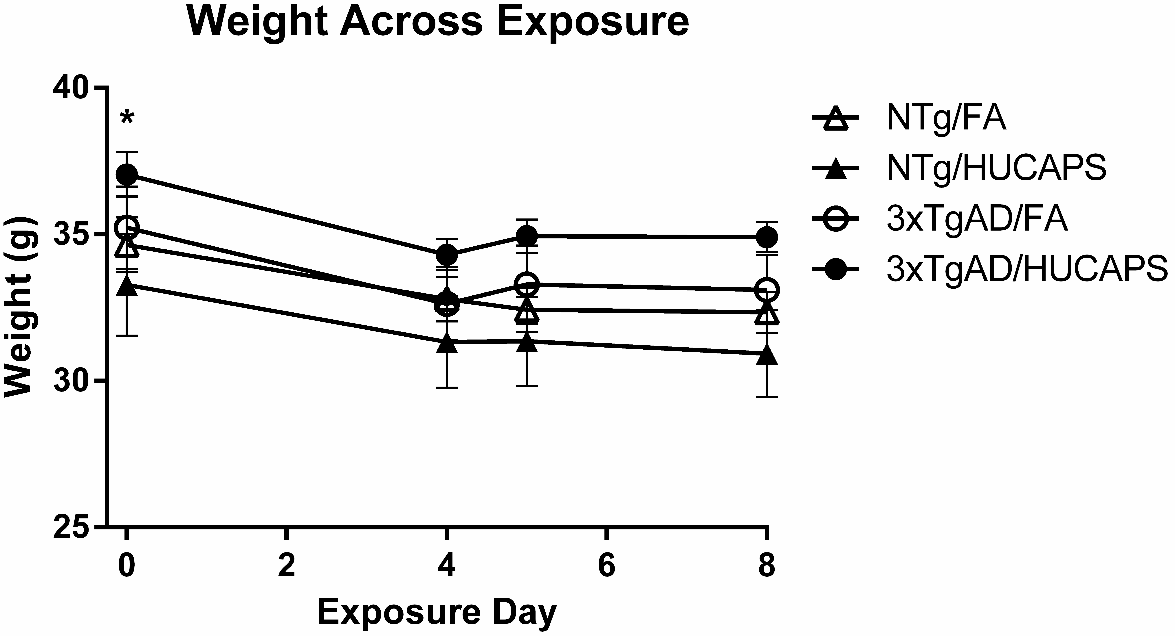


|  | NTg/FA | NTg/HUCAPS | 3xTgAD/FA | 3xTgAD/HUCAPS |
| --- | --- | --- | --- | --- |
| Pre-exposure weight (g) | 34.9 ± 3.1 | 33.6 ± 5.6 | 35.3 ± 4.9 | 37.1 ± 2.5 |
| Average weight across exposure (g) | 32.5 ± 2.4 | 31.2 ± 5.0 | 33.0 ± 4.2 | 34.7 ± 1.7 |
| Percent loss during exposure | 6.6 ± 2.4 | 6.9 ± 2.1 | 6.3 ± 2.1 | 6.2 ± 2.9 |

Exposure day 0 is average weight pre-exposure. t-tests were performed comparing all (pre-) exposure day weights. (*) indicates significant difference from other exposure days. Table shows means ± SD.

**Supplemental 12:** MPPD inputs and scaling adjustments

Bolded inputs correspond to allometrically adjusted parameters which were: respiratory rate (RR), tidal volume (V_T_), and minute ventilation (MV).

Experimental mice mean body weight (BW) = 35.2 g

Allometric Adjustments:

Respiratory rate = 82 * BW^-0.287^(kg) [5]

RR= 82 * 0.0352^-0.287^ = 214 breaths/min

Tidal Vol (ml) = 0.0074*Body weight (g) [6]

V_T_ = 35.2 g * 0.0074 = 0.260 ml

Minute Ventilation = 8.72 * BW (in kg)^0.704^*60 [7]

MV = 8.72 * 0.0352^0.704^*60 = 49.6 ml/min = 0.0496 L/min

**MPPD Inputs:**

Avg weight of mice prior to behavior (just ones used for analysis) = 35.2 g

Utilized the mouse: B6C3F1 model (more similar growth rate curves as our mice)

Default functional residual capacity = 0.3 ml

Default upper respiratory tract volume = 0.0322 ml

Aerosol:

Default density = 1.0 g/cm^3^

Default aspect ratio = 1 (spherical)

CMD diameter = 0.079 um (single mode)

GSD (diameter) = 1.47

Exposure Scenario: Constant exposure

Body orientation: on stomach

Aerosol concentration: 0.0571 mg/m3

**RR:** 214 breaths/min

**V_T_:** 0.260 ml

Default inspiratory fraction: 0.5

Default pause fraction: 0

Whole body exposure

**Daily Deposited Dose Calculation:**

Daily deposited dose = MV (L/min) x Duration(Min) x Mass concentration(ug/m^3^) x Deposited fraction (MPPD)

Duration = 4 hr*1day*60min/hr = 240 min

Daily Deposited dose (µg) = 0.0496 L/min * 240 min * 57.1 ug/m^3^ * Dep fraction * 1m^3^/1000L

| **Region** | **Dep Fraction** | **Daily Deposited Dose (ng)** |
| --- | --- | --- |
| Head | 0.1463 | 99 |
| TB | 0.0529 | 36 |
| Pulmonary | 0.1958 | 133 |
| Total | 0.3950 | 268 |

**Supplemental 13:** Mouse body weight measured prior to exposure


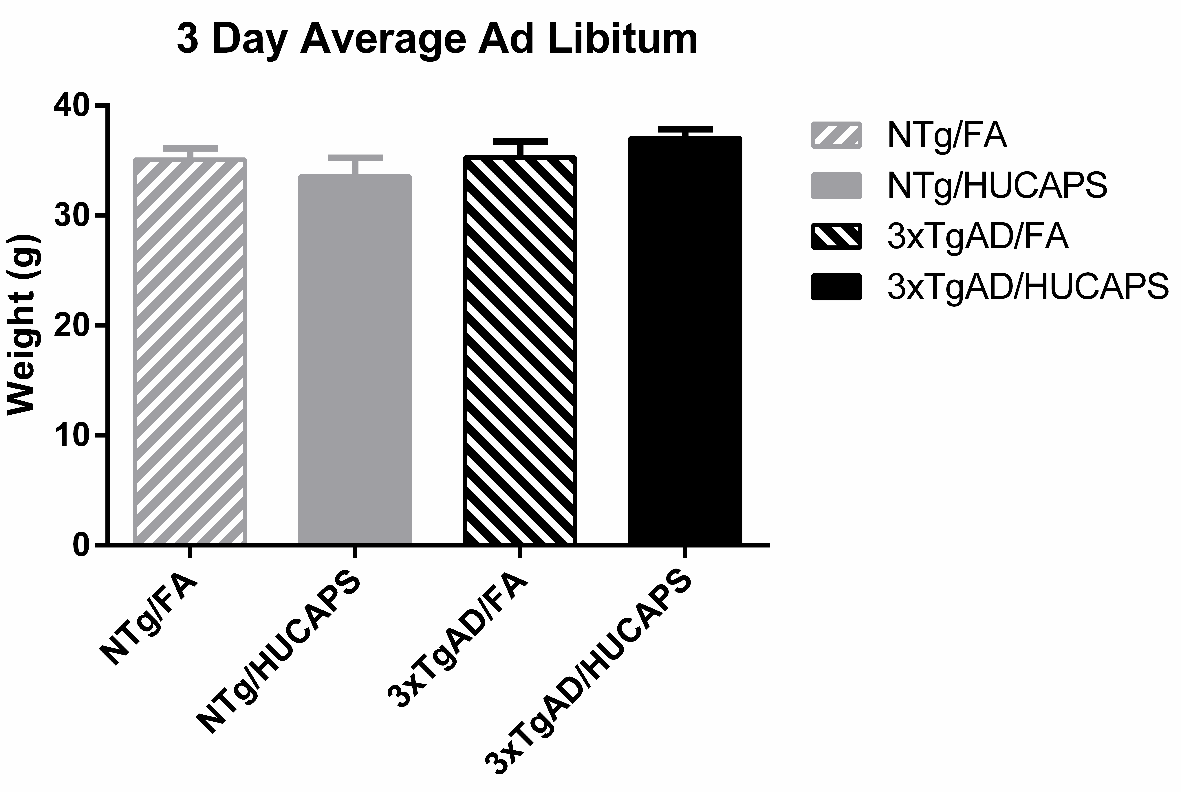


*Ad libitum* body weight measured prior to exposure. Means ± SE.

**References:**

1. Kecorius S, Madueno L, Londahl J, Vallar E, Galvez MC, Idolor LF, et al. Respiratory tract deposition of inhaled roadside ultrafine refractory particles in a polluted megacity of South-East Asia. Sci Total Environ. 2019;663:265-74; doi: 10.1016/j.scitotenv.2019.01.338.

2. Menache MG, Hanna LM, Gross EA, Lou SR, Zinreich SJ, Leopold DA, et al. Upper respiratory tract surface areas and volumes of laboratory animals and humans: considerations for dosimetry models. J Toxicol Environ Health. 1997;50 5:475-506; doi: 10.1080/00984109708984003.

3. Pozarska A, Rodriguez-Castillo JA, Surate Solaligue DE, Ntokou A, Rath P, Mizikova I, et al. Stereological monitoring of mouse lung alveolarization from the early postnatal period to adulthood. Am J Physiol Lung Cell Mol Physiol. 2017;312 6:L882-L95; doi: 10.1152/ajplung.00492.2016.

4. Wiebe BM, Laursen H. Human lung volume, alveolar surface area, and capillary length. Microsc Res Tech. 1995;32 3:255-62; doi: 10.1002/jemt.1070320308.

5. Piccione G, Caola G, Mortola JP. Scaling the daily oscillations of breathing frequency and skin temperature in mammals. Comp Biochem Physiol A Mol Integr Physiol. 2005;140 4:477-86; doi: 10.1016/j.cbpb.2005.02.010.

6. Guyton AC. Measurement of the respiratory volumes of laboratory animals. Am J Physiol. 1947;150 1:70-7; doi: 10.1152/ajplegacy.1947.150.1.70.

7. Lindstedt SL, Schaeffer PJ. Use of allometry in predicting anatomical and physiological parameters of mammals. Lab Anim. 2002;36 1:1-19; doi: 10.1258/0023677021911731.
